# Primary adrenal nodular lymphocyte-predominant Hodgkin lymphoma: A case report and review of the literature

**DOI:** 10.3892/ol.2014.2289

**Published:** 2014-06-26

**Authors:** JINGJING WANG, JIN’AN MA, CHUNHONG HU, DAIQIANG LI, XIAOLING SHE

**Affiliations:** 1 Department of Oncology, The Second Xiangya Hospital of Central South University, Changsha, Hunan 410011, P.R. China; 2 Department of Pathology, The Second Xiangya Hospital of Central South University, Changsha, Hunan 410011, P.R. China

**Keywords:** nodular lymphocyte-predominant Hodgkin lymphoma, adrenal gland, histopathology

## Abstract

Nodular lymphocyte-predominant Hodgkin lymphoma (NLPHL) is a subtype of Hodgkin lymphoma (HL), and is a rare disease manifestation in the adrenal gland, which is difficult to be diagnosed and treated. In the present study, we report a case of primary adrenal NLPHL in a 36-year-old male patient. The patient was without specific clinical signs and the definitive diagnosis was achieved by histological study. The patient underwent a laparoscopic left adrenalectomy and chemotherapy regimen of doxorubicin, bleomycin, vinblastine and dacarbazine (ABVD). There is no standard treatment for adrenal NLPHL and therefore, treatment is based on that for other types of NLPHL, which includes radiotherapy and ABVD chemotherapy. Given the rarity of this disease, there are limited experiences with regard to its diagnosis and treatment. This study is useful for the differential diagnosis and treatment of adrenal masses.

## Introduction

The majority of adrenal lesions are benign adrenal cortical adenomas. However, nodular lymphocyte-predominant Hodgkin lymphoma (NLPHL) is a rare disease manifestation in the adrenal gland. NLPHL is a subtype of Hodgkin lymphoma (HL), which represents ~5% of HLs and involves peripheral lymph nodes. NLPHL is a ‘benign’ disease which is characterized by an indolent course (1,2). Mortalities due to NLPHL are rare, however treatment toxicities and varsious secondary malignancies may contribute to the overall morality rate. Secondary aggressive non-Hodgkin lymphoma may occur in ~7–14% of NLPHL cases, but the majority of NLPHL patients present at an early stage and have a favourable prognosis (3–6). The peripheral and most commonly the cervical regions are involved (1,2). NLPHL has a peak incidence in the fourth decade of life with a significant male predominance (75%). B symptoms, extranodal involvement and bulky disease are particularly uncommon (7). We report a unique case of NLPHL affecting only the adrenal gland in a patient who was successfully diagnosed by histological examination, and treated with surgery and a chemotherapy regimen of doxorubicin, bleomycin, vinblastine and dacarbazine. This study was approved by the ethics committee of the Second Xiangya Hospital of Central South University (Changsha, China). Patient provided written informed consent.

## Case report

### Case presentation

**Figure 1:**
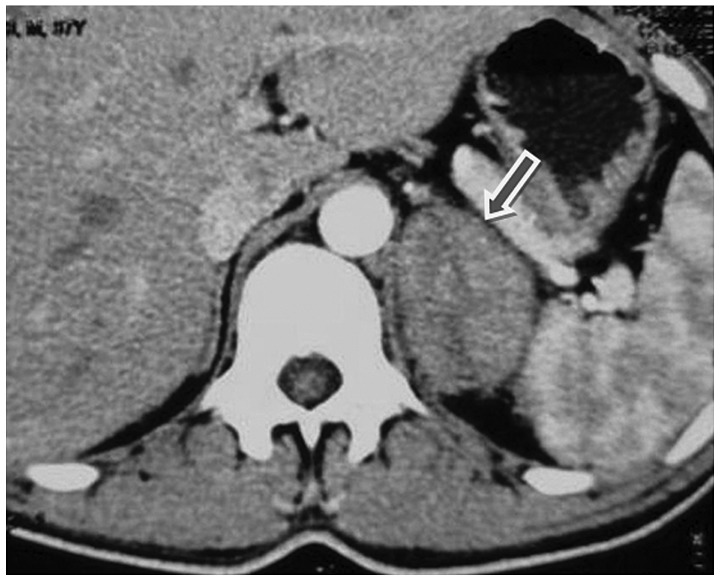
Abdominal computed tomography of the patient with primary adrenal nodular lymphocyte-predominant Hodgkin lymphoma. A soft tissue mass was observed in the left adrenal gland (arrow), measuring 4.5×5.5 cm, with a less uniform density and clear borders.

A 36-year-old male presented at The Second Xiangya Hospital of Central South University (Changsha, China) with high fever and weight loss lasting for 10 days, as well as a left adrenal mass that had been identified one week previously. The patient had no history of hypertension and physical examination showed no abnormalities. A total body computed tomography (CT) scan revealed the presence of a large left adrenal mass measuring 4.5×5.5 cm in size (Fig. 1). No obvious abnormalities in the right adrenal gland, retroperitoneal area and other parts of the body were identified by CT. Bone marrow aspiration and biopsy were normal. Plasma metanephrine, plasma renin activity, serum aldosterone, dehydroepiandrosterone and basic metabolic panel test results were all normal. Routine hematological parameters and liver and kidney function tests were normal. Hepatitis B and C and human immunodeficiency virus serology test results were negative. In addition, Epstein-Barr virus (EBV) serology showed negativity for immunoglobulin M (IgM) and IgG antibodies. The patient subsequently underwent laparoscopic left adrenalectomy.

### Pathology

**Figure 2:**
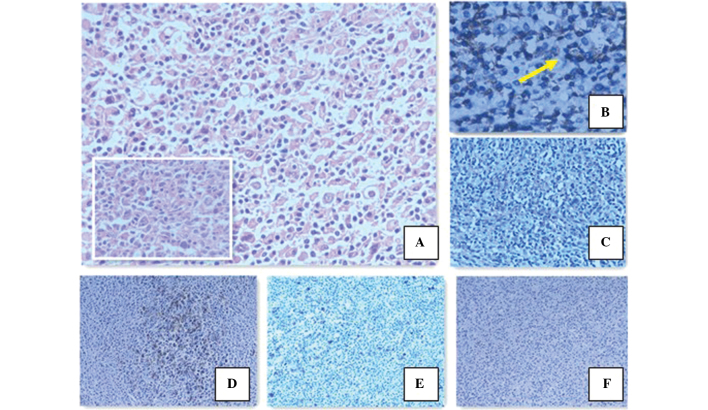
Histological and immunohistochemical analysis of the pathological tissue of the patient with primary adrenal nodular lymphocyte-predominant Hodgkin lymphoma. (A) Hematoxylin and eosin staining of the tumor tissue (magnification of main image, ×200; magnification of the smaller image, ×400). (B) CD3^+^ T-cell rosettes surrounding the tumor cells as indicated by the arrow (immunostaining; magnification, ×200). (C) EMA^+^ LP cells (immunostaining; magnification, ×200). (D) CD20^+^ LP cells (immunostaining; magnification, ×100). (E) A few CD15^+^ LP cells (immunostaining; magnification, ×100). (F) CD30^−^ LP cells (immunostaining; magnification, ×100).

The adrenalectomy specimen measured 5.5×4.0×4.0 cm. Microscopic examination revealed tumors of lymphoid-hematopoietic tissues in the adrenal tissue, which destroyed the adrenal gland and formed a mass in the retroperitoneum. Morphologically, the majority of atypical cells resembled lymphocyte-predominant (LP) cells. Immunohistochemical analysis showed that the tumor cells were negative for cluster of differentiation 30 (CD30) and EBV-encoded small RNAs (EBERs), and positive for CD20, paired box protein 5, CD79a, octamer binding protein 2 and epithelial membrane antigen (EMA). Additionally, very few cells were positive for CD15. The tumor cells were surrounded by T cells, which were positive for CD3 and CD57 (Fig. 2). According to the immunohistochemistry results, the patient was diagnosed with primary adrenal NLPHL, stage IB according to the World Health Organization (WHO) (8).

### Postoperative treatment

Systemic positron emission tomography (PET)-CT examination was conducted following surgery, and there were no obvious abnormalities. Postoperative adjuvant chemotherapy was administered; the patient received four cycles of doxorubicin, bleomycin, vinblastine and darcarbazine (ABVD) regimen. This comprised 25 mg/m^2^ doxorubicin (day 1 and 14), 10 mg/m^2^ bleomycin (day 1 and 14), 6 mg/m^2^ vinblastine (day 1 and 14) and 375 mg/m^2^ dacarbazine (day 1 and 14), to be repeated every four weeks. The patient has been followed up for 16 months from the end of chemotherapy, with a stable condition and no recurrence.

## Discussion

According to the present WHO classification, HLs comprise two disease entities: NLPHL and classical HL (cHL) (8). cHL represents ~95% of HLs, while NLPHL is rare, representing ~5%. NLPHL has a male predominance and typically presents in middle-aged individuals. NLPHL frequently presents as localized disease and has a good outcome. The ten year overall survival rate for early-stage patients is ~85–100% (3,4). Approximately 20–25% of NLPHL patients present with an advanced stage of the disease. Xing et *al* (9)demonstrated that the ten year overall survival for patients with advanced NLPHL is 86%. The most common sites of involvement are the peripheral lymph nodes, and disease manifestation in the adrenal gland is rare. To the best of our knowledge, only three cases of primary adrenal HL have been reported worldwide, two of which were adrenal cHL (10,11) and one of which was composite lymphoma of NLPHL and cHL concurrently affecting the same lymph nodes (12).

Ultrasound, CT and magnetic resonance imaging have become the preferred methods for identifying adrenal tumors. PET-CT is not only able to distinguish between benign and malignant tumors of the adrenal gland in order to make a diagnosis, but also plays a significant role in the staging, evaluation of therapeutic effects and prediction of patient prognosis in HL (13).

NLPHL is characterized by a nodular or nodular and diffuse growth pattern. The neoplastic cells in NLPHL have been known as lymphocytic and histiocytic cells, LP cells or ‘popcorn’ cells, which are embedded in a nodular non-neoplastic background mainly composed of small B lymphocytes and some CD57^+^ T cells. The immunophenotype of NLPHL is useful for the diagnosis. LP cells are positive for B-cell markers including CD19, CD20, CD22 and CD79a, in addition to CD45 and EMA; however, they lack expression of CD15 and CD30, which are the characteristic markers for cHL. In the background, CD3^+^ or CD57^+^ T cells typically surround the LP cells, forming what are commonly referred to as T-cell rosettes (14). NLPHL is not associated with EBV infection, so EBERs are negative in LP cells. The tumor cells of NLPHL originate from B cells characterized by somatic mutations in the variable domain of the rearranged Ig heavy chain genes and the expression of B-cell markers. The morphological and immunohistochemical features in the present case were consistent with the diagnosis of NLPHL.

Patients with NLPHL are mainly treated with radiotherapy and chemotherapy (15,16). Surgery is mainly used to obtain the pathological tissue and diagnosis, and does not affect the prognosis (6).

In early-stage, limited NLPHL cases (I and II stage) treatment with radiotherapy alone has been recommended in the past. Clinical studies on 202 patients in Australasia showed that the 15-year overall survival (OS) rate was 83% and the freedom from progression rate was 82% in patients with I-II stage LPHL treated with radiotherapy alone (17). Another study, conducted in Germany, showed that of 131 patients, 98% of patients with stage IA LPHL achieved complete remission following radiotherapy (18). The suggested cumulative dose is 30–36 Gy (19). However, more recent studies have found that treating limited-stage NLPHL with ABVD-like chemotherapy for two cycles followed by radiotherapy may improve outcome compared with the use of radiation alone (3,20). A retrospective study analyzed 88 patients with limited stage NLPHL and the results showed a marked improvement in the 10-year time to progression (98 vs. 76%; P=0.0074), progression-free survival (91 vs. 65%; P=0.0024) and OS (93 vs. 84%; P=0.074) for patients treated with ABVD followed by radiotherapy compared with those treated with radiotherapy alone (3).

Patients with early-stage NLPHL who have received ABVD chemotherapy and/or radiotherapy have a good prognosis; studies have shown that the 10-year overall survival rate is >90%, even up to 100% (1,2). Patients who seek to achieve long-term survival must consider the side effects of treatment and treatment-related mortality. A recent study of 405 patients showed that the 12-year overall survival rate was 94% among those receiving treatment with ABVD alone, as compared with 87% among those receiving radiation therapy (P=0.04). It was suggested that among patients with early-stage HL, ABVD therapy alone was associated with a higher rate of overall survival compared with radiation therapy, owing to a lower rate of death from other causes (21). However, a number of studies have suggested a watch-and-wait strategy as a potentially curative approach after initial lymph node surgery, particularly for children (2,22,23). The optimal treatment for limited-stage NLPHL is unclear, and very few cases of adrenal NLPHL have been reported. As a result, there is no standard treatment for adrenal NLPHL and a suitable treatment can only be determined based on that of other types of NLPHL. In order to reduce treatment-related toxicity, the patient in the present case was only treated with adjuvant chemotherapy after surgery. The invasion site of the tumor presented in this case was the kidney. In order to prevent damage to the kideny from radiotherapy and to reduce treatment-related toxicity, the patient was treated only with ABVD adjuvant chemotherapy following a laparoscopic left adrenalectomy.

The current recommendation for advanced-stage NLPHL consists of standard cHL protocols, with ABVD chemotherapy and combined therapy including radiotherapy (3).

The LP cells in NLPHL express high levels of CD20 antigen, rituximab, which has emerged as a potential treatment option for NLPHL. Ekstrand *et al* (24) conducted a phase 2 trial where 22 patients with LPHL (untreated and previously treated, stages I-III) were treated with rituximab. The overall response rate was 100%. The German Hodgkin Lymphoma Study Group reported an overall response rate (ORR) of 94% in NLPHL patients administered with rituximab, including eight patients who achieved complete remission (CR) and six who achieved partial remission (PR) (25). It was suggested that rituximab was highly effective in relapsed and refractory NLPHL. In another phase 2 trial, newly diagnosed stage IA NLPHL patients treated with rituximab were investigated. Among the 28 evaluable patients, the ORR was 100%, 85.7% patients achieved CR and 14.3% achieved PR, and the 43-month OS rate was 100% (14). Treatment results with rituximab appear inferior compared with radiotherapy and combined-modality approaches in early-stage patients. The 2012 National Comprehensive Cancer Network guidelines recommend rituximab for the treatment of NLPHL of stage Ib or greater (26).

Primary adrenal NLPHL is extremely rare, and pathological analysis and immunohistochemical examination are key in achieving a diagnosis. Due to the rarity of NLPHL, there have been few prospective studies, and as a result, a wide range of management approaches are available. Thus, the optimal management strategy for NLPHL is unclear. Patients with limited stage NLPHL treated with ABVD chemotherapy followed by radiotherapy have more successful outcome compared with those treated with radiotherapy alone. The current recommendation for advanced-stage NLPHL consists of standard cHL protocols, with ABVD chemotherapy and combined therapy including radiotherapy and rituximab. Rituximab has emerged as a potential treatment option for NLPHL. The optimal treatment of NLPHL in the era of targeted therapy should be further explored. Further studies, particularly long-term, prospective randomized clinical trials are required to improve the diagnostic techniques and the optimal treatment of NLPHL. Additionally, the differential diagnosis of adrenal tumors should be considered.
